# Protocol for a mixed-methods study to develop and feasibility test a digital system for the capture of patient-reported outcomes (PROs) in patients receiving chimeric antigen receptor T-cell (CAR-T) therapies (the PRO-CAR-T study)

**DOI:** 10.1136/bmjopen-2024-085392

**Published:** 2024-03-29

**Authors:** Sarah E Hughes, Christel McMullan, Olalekan Lee Aiyegbusi, Karen Shaw, Francesca Kinsella, Paul Ferguson, Foram Khatsuria, David Burns, Lester Pyatt, John Ansell, Evelyn Chakera, Julie Richardson-Abraham, Alastair K Denniston, Elin Haf Davies, Charles Craddock, Melanie Calvert

**Affiliations:** 1 Centre for Patient Reported Outcome Research, Institute of Applied Health Research, University of Birmingham, Birmingham, UK; 2 National Institute of Health and Care Research (NIHR) Blood and Transplant Research Unit (BTRU) in Precision Cellular Therapeutics, Birmingham, UK; 3 NIHR Applied Research Collaboration (ARC) West Midlands, Birmingham, UK; 4 Birmingham Health Partners Centre for Regulatory Science and Innovation, University of Birmingham, Birmingham, UK; 5 NIHR Birmingham Biomedical Research Centre, Birmingham, UK; 6 University Hospitals Birmingham NHS Foundation Trust, Birmingham, UK; 7 University of Birmingham, Birmingham, UK; 8 Patient Author, Birmingham, UK; 9 Academic Unit of Opthalmology, Institute of Inflammation and Ageing, University of Birmingham, Birmingham, UK; 10 Aparito Ltd, Wrexham, UK; 11 Centre for Clinical Haematology, University Hospitals Birmingham NHS Foundation Trust, Birmingham, UK

**Keywords:** Quality of Life, Patient-Centered Care, Gene therapy, Patient Reported Outcome Measures, HAEMATOLOGY, eHealth

## Abstract

**Introduction:**

Chimeric antigen receptor (CAR) T-cell therapies are novel, potentially curative therapies for haematological malignancies. CAR T-cell therapies are associated with severe toxicities, meaning patients require monitoring during acute and postacute treatment phases. Electronic patient-reported outcomes (ePROs), self-reports of health status provided via online questionnaires, can complement clinician observation with potential to improve patient outcomes. This study will develop and evaluate feasibility of a new ePRO system for CAR-T patients in routine care.

**Methods and analysis:**

Multiphase, mixed-methods study involving multiple stakeholder groups (patients, family members, carers, clinicians, academics/researchers and policy-makers). The intervention development phase comprises a Delphi study to select PRO measures for the digital system, a codesign workshop and consensus meetings to establish thresholds for notifications to the clinical team if a patient reports severe symptoms or side effects. Usability testing will evaluate how users interact with the digital system and, lastly, we will evaluate ePRO system feasibility with 30 CAR-T patients (adults aged 18+ years) when used in addition to usual care. Feasibility study participants will use the ePRO system to submit self-reports of symptoms, treatment tolerability and quality of life at specific time points. The CAR-T clinical team will respond to system notifications triggered by patients’ submitted responses with actions in line with standard clinical practice. Feasibility measures will be collected at prespecified time points following CAR T-cell infusion. A qualitative substudy involving patients and clinical team members will explore acceptability of the ePRO system.

**Ethics and dissemination:**

Favourable ethical opinion was granted by the Health and Social Care Research Ethics Committee B(HSC REC B) (ref: 23/NI/0104) on 28 September 2023. Findings will be submitted for publication in high-quality, peer-reviewed journals. Summaries of results, codeveloped with the Blood and Transplant Research Unit Patient and Public Involvement and Engagement group, will be disseminated to all interested groups.

**Trial registration number:**

ISCTRN11232653.

**STRENGTHS AND LIMITATIONS OF THIS STUDY**

- Data for the study are strengthened by inclusion of multiple stakeholder groups, including patients, carers, healthcare professionals, academics and policy-makers.
- Consensus methods will be deployed at all stages of intervention development, ensuring the views of relevant groups are incorporated into the system’s design.
- Applying the fit between individuals, task and technology framework and the use of validated questionnaires will enable theory-driven interrogation of electronic patient-reported outcomes system usability.
- Semistructured interviews in the qualitative substudy will permit in-depth exploration of system feasibility and acceptability with patients and clinicians.
- Duration of feasibility study means monitoring of chimeric antigen receptor-T patients beyond 12 months postinfusion will not be undertaken.

## Introduction

Chimeric antigen receptor (CAR) T-cell therapies are innovative therapies for treatment of haematological malignancies. Using T-cells that have been genetically engineered to redirect these cells’ cytotoxic specificity towards tumour cells, CAR T-cell therapy has shown promising results in terms of the durability of remission, with a recent meta-analysis reporting a complete response in 54.4% of patients who received CD19 CAR T-cells across 27 studies.^1 2^ However, these encouraging findings are tempered with significant safety concerns arising from unique treatment-related toxicities that can be life-threatening.^3^

Cytokine release syndrome (CRS) and immune effector cell-associated neurotoxicity syndrome (ICANS) are the most commonly occurring toxicities following CAR-T-cell infusion. CRS is a systemic inflammatory response caused by cytokines released by the infused T-cells. Symptoms are numerous and heterogeneous, affecting multiple organ systems and, in severe cases, can lead to organ failure.^4 5^ CRS of any grade is estimated to present in a significant proportion of CAR-T patients with estimates ranging from 37% to 93% in patients with lymphoma and 77% to 93% of leukaemia patients.^3^ ICANS can occur concurrently or after CRS. Signs and symptoms can include confusion, tremor, expressive aphasia and seizures.^2 5 6^

Due to the unique toxicity profile of CAR-T cell therapies, patients receiving these novel treatments require intensive monitoring by their clinical team. Patient-reported outcomes (PROs) are reports of a person’s health (ie, symptoms, functioning, quality of life) that come directly from the individual without interpretation by a clinician or anyone else.^7^ PROs can prove a useful complement to clinician observation. For example, a randomised controlled trial (RCT) of patients with cancer receiving chemotherapy found that remote monitoring using PROs improved survival, reduced hospital admissions and improved patients’ quality of life compared with usual care.^8^ Furthermore, evidence suggests clinicians may under-report the incidence and severity of adverse event-related toxicities. A systematic review found poor-to-moderate associations between analogous clinician-reported Common Terminology Criteria for Adverse Events (CTCAE) and PROs, regardless of the PRO measure used.^9^ PROs may also be useful in clinical practice to inform decision-making around treatment and care, support communication between the patient and their healthcare team, and aid self-management.^10 11^ While PRO data can help to ensure that patients’ views about their treatment and side effects remain at the forefront of care, they are currently under-reported in clinical trials of CAR-T products and there is limited literature exploring the use of PROs for monitoring of CAR-T-related adverse events and quality of life in clinical settings, particularly in a UK context.^12^

The field of precision cellular therapy is forecast to undergo rapid expansion in the coming decade and there is an urgent need for evidence-based tools, coproduced with patients, to better support the growing number of individuals with haematological malignancies who will receive these potentially curative treatments. The novelty of CAR-T cell therapies and the unique, potentially severe toxicities associated with these treatments mean effective symptom monitoring is key to maximising treatment benefit, ensuring patient safety and reducing care costs. Remote monitoring using electronic PROs (ePRO) systems could enable patients to be supported at home or otherwise away from the clinic and hospital ward. Findings from a recent pilot study have suggested that longitudinal PRO data capture is both feasible and acceptable for use with CAR-T cell therapy patients.^6^

The PRO-CAR-T study aims to develop and assess the feasibility of an ePRO system for patients receiving CAR-T cell therapies and is aligned with research priorities highlighted in the UK Stem Cell Strategic Forum’s report ‘A 10-year vision for stem cell transplantation and cellular therapies’.^13^ Development work will include concept elicitation and development of the conceptual framework and measurement strategy that will underpin the PRO-CAR-T system and codesign of the system including the format of alert notifications to the clinical team (when symptoms of concern are reported by the patient). We will evaluate the feasibility of the digital system with CAR-T therapy patients in a clinical setting to help inform the design of a full-scale RCT evaluating the effectiveness of ePRO remote monitoring in patients receiving CAR T-cell therapies.

## Aims and objectives

This mixed-methods multiphase study aims to develop a new digital platform to capture PROs for remote monitoring of symptoms, side effects and health-related quality of life in patients with haematological malignancies receiving CAR T-cell therapies and assess its feasibility for use in the UK’s National Health Service (NHS).

The key objectives are as follows:

1. To develop a conceptual framework for measurement that will underpin the PRO-CAR-T digital system.
2. To identify and shortlist candidate PRO measures and map these to the digital system’s conceptual framework for measurement.
3. To select, through a consensus-building process with stakeholders, the PRO measures for inclusion in the digital platform.
4. To identify items in the included PRO measures that assess symptoms and other constructs of clinical relevance or concern (eg, severe side effects, treatment tolerability, quality of life) that require notifications to the clinical team through system alerts.
5. To understand stakeholder needs in relation to PRO-based alert management within routine CAR-T care and to codevelop the alert functionality for the digital platform.
6. To test the usability of the new digital system to ensure the platform is effective, efficient and perceived as satisfactory by end users.
7. To assess feasibility and acceptability of the new digital system to capture PROs within a routine CAR-T clinical setting.

## Methods and analysis

### Design

**Figure 1:**
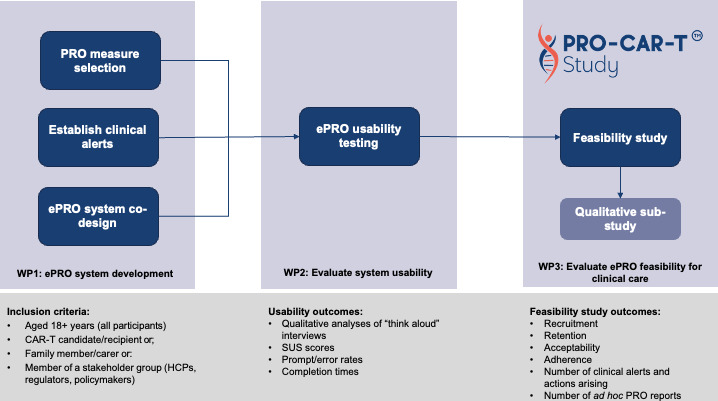
Study flow diagram for the PRO-CAR-T study. CAR-T, chimeric antigen receptor T-cell; ePRO, electronic patient-reported outcome; HCP, healthcare professional; PRO, patient-reported outcome; SUS, System Usability Scale.

The PRO-CAR-T study comprises three, sequential work packages (WP) or phases, each building on the former. WP 1 involves development of the digital intervention (ePRO system); WP2 involves building and testing system usability; and WP3 involves evaluating the feasibility of the digital system when it is deployed in a clinical setting and includes a qualitative substudy (figure 1).

### The Aparito Atom5™ platform

The PRO-CAR-T ePRO system will be deployed using the Atom5™ digital platform developed by Aparito. Atom5™ is a configurable, multilingual, disease-agnostic digital platform for remote patient monitoring. It has several capabilities that make it ideally suited to patient monitoring following CAR-T cell therapy, including the capability to track symptoms and quality of life with the use of ePROs, deliver tailored notifications and alerts with links to relevant support services and to convey data in real-time to healthcare professionals (HCPs) and researchers. The platform comprises a patient-facing desktop portal accessed via an internet browser, patient-facing app (Android and iOS compatible), clinician/study staff-facing desktop web portal and data analytics dashboard. Each deployment is GxP compliant. Aparito is accredited for Quality Management System, ISO 13485 and Information Security Management System ISO 27001.

### Population and setting

CAR-T patients in the UK who are managed by the Birmingham Centre for Cellular Therapy and Transplant at Queen Elizabeth Hospital, University Hospitals Birmingham NHS Foundation Trust will be invited to participate in this study. Depending on the specific WP, other participant groups will include family members, HCPs, academics/researchers, regulators, policy-makers and industry representatives.

### WP 1: ePRO system development

The PRO-CAR-T digital system specification will be coproduced through a series of workshops and/or individual interviews (depending on participant preference) involving patients and their family members, HCPs, academics/researchers, policy-makers and industry representatives. First, a conceptual framework for measurement and shortlist of candidate PRO measures will be generated from a systematic search and rapid review of the published literature.^14^ The conceptual framework will provide a content map describing the expected relationships between constructs to be measured by the PRO-CAR-T system and the items/domains of a PRO measure.^15^ Next, a Delphi review panel involving up to 30 participants will select PRO measures for inclusion in the PRO-CAR-T system from the list of shortlisted measures. The Delphi study will comprise two rounds: (1) an online survey followed by (2) an online workshop to reach consensus on the PRO measures to be included.

Last, we will hold a series of workshops (or individual interviews depending on participant preference) to (1) ascertain the essential and desirable components of an ePRO system (codesign) and (2) establish those items from the included PRO measures that, if reported by a CAR-T patient, would be of sufficient clinical concern to require notification to the patient’s clinical team. We will explore codesign elements with participants including key stakeholders’ requirements for symptom reporting, information and communication mapped against the patient journey. Findings will ensure the design of the PRO-CAR-T system is grounded in the needs and expectations of users.^16^ The codesign approach is based on ISO standards for human-centred design for interactive systems (European Committee for Standardisation 2010), ISPOR guidance on ePRO systems, and the user innovation management method.^16–18^ To establish alerting requirements, workshops and interviews will explore stakeholders preferences for the format of alert notifications, establish scoring thresholds and arising actions, and map the end-to-end clinical workflow that will occur within the Atom5 environment. We will invite up to 30 individuals to take part from the following participant groups: CAR-T patients and their family members, HCPs and academics/researchers.

For each component of intervention development (WP 1), we will use descriptive statistics to analyse quantitative survey data (overall and per participant group) using Excel, STATA (v18) or SPSS (v29). For qualitative analyses, we will apply thematic analysis to inductively code free text survey responses and workshop transcripts.^19^ Two researchers will independently code a subset of the transcripts to cross check the coding strategy and data interpretation and the Consolidated Criteria for Reporting Qualiative Research (COREQ) checklist will be used as a guideline for reporting qualitative methods and findings.^20^ Deductive frameworks will support coding of data to develop the system specification and *a priori* criteria, based on Murphy *et al* and Williamson *et al*, will be applied to establish consensus on the PRO measures to be included in the PRO-CAR-T digital system.^21 22^

### WP 2: usability testing

Usability testing refers to the ‘formal assessment of the extent to which interaction with a product or system is effective, efficient and perceived as satisfactory by users’ (Aiyegbusi, p326)^23^ and typically involves observation of end users interacting with the digital tool as they complete a series of tasks. CAR T-cell therapy patients and HCPs working in clinical settings offering CAR T-cell therapies will participate in a cognitive interview to evaluate the usability of the PRO-CAR-T system. A sample of 20–30 patients and HCPs will take part in two cycles of testing. The PRO-CAR-T system in Atom5™ will be refined iteratively after each test cycle until no new problems are encountered by participants. The usability test sessions will be conducted remotely using videoconferencing software. Test sessions will be recorded and transcribed verbatim for analysis. During the test session, patient participants will be asked to download Atom5™, access the PRO-CAR-T system and navigate the system to complete the PRO measures. While navigating the system, patients will be asked to ‘think-aloud’ to describe their thought process to the researcher. Patients will be informed that they do not have to answer the questions about their symptoms or quality of life truthfully, that the emphasis of testing is to evaluate the PRO-CAR-T system’s ease of use from the user perspective, and that any data they enter will not be saved nor used to inform their care. HCPs will log in to the PRO-CAR-T clinical dashboard and complete specific tasks (eg, register a patient, respond to a clinical alert) while thinking aloud. In the case of HCPs, fictitious patient data will be used. On completing the tasks, participants will be asked to provide feedback relating to their experience using the digital system including ways the user experience could be improved.

A semistructured topic guide will be used to structure the interview and participants will also complete the System Usability Scale (SUS), a 10-item questionnaire providing an assessment of usability.^24^ SUS items are scored on a 5-point rating scale ranging from ‘strongly disagree’ to ‘strongly agree’. Raw scores are converted to 0–100 score with higher scores indicating greater usability. A score greater than 80 is considered evidence of above average user experience.^25^ The psychometric properties of the SUS have been evaluated extensively and the scale has been shown to have good internal consistency reliability (Cronbach’s alpha=0.83–0.91) and concurrent validity with other measures of usability.^26^ A multimethod approach to usability (ie, usability metrics, self-completed surveys and cognitive interviewing techniques) was selected to counter positive recall bias associated with think-aloud techniques.^23^

Data analysis will be guided by the fit between individuals, task and technology (FITT) framework to describe usability on three dimensions: (1) task-technology fit; (2) individual-technology fit and (3) individual-task fit.^27^ Developed specifically for use in healthcare, the FITT framework enables developers to understand the relationship between users, tasks and technology and the factors affecting optimal device use.^28^ Descriptive statistics will be used to describe the demographic characteristics of the study sample, SUS scores, prompt/error rates and task completion times. Excel and SPSS or STATA statistics software will be used for all statistical analyses.

Cognitive ‘think-aloud’ interviews will be qualitatively analysed using thematic analysis and the framework method.^29^ A deductive coding framework will be used to code data according to the FITT model and inductive coding will identify additional concepts in the data. NVivo (V.14) qualitative data analysis software will be used to manage and code the transcribed interview data. Two researchers will independently code a subset of the data to cross check the coding strategy and data interpretation. Problems with the ePRO system identified during usability testing will be logged, discussed with Aparito and a system change request generated. Refinements to the system will be made before commencing the next test cycle.

### WP 3: feasibility study

The PRO-CAR-T feasibility study is a single-centre study of the feasibility and acceptability of the new ePRO system when deployed in the UK’s NHS. 30 CAR-T patients will be recruited consecutively to use the PRO-CAR-T system (ie, in addition to usual care). Patients who consent to participate will be onboarded to the system prior to infusion. During the baseline visit, participants will download the Atom5 app to their mobile device, give their demographic details including age, sex at birth, ethnicity, cancer diagnosis, CAR T-cell therapy, and self-reported experience with technology and complete the PRO measures for the first time. PRO measures will be completed subsequently via the PRO-CAR-T system at prespecified intervals (measures and frequency of assessments will be established in WP 1). We will follow participants for 12 months from date of infusion (day 0) to understand the feasibility and acceptability of longitudinal collection of PROs for CAR-T patients, their families and HCPs. The findings will be used to inform a future, definitive RCT to assess the effectiveness of remote symptom monitoring using the digital system alongside usual care compared with usual care alone.

#### Sample size justification

There is no definitive guidance relating to sample sizes in pilot and feasibility studies with a heuristic of 30 (range=10–40) patients generally applied. Therefore, no formal sample size calculation has been performed for the feasibility study. To allow for a 15% attrition rate, a minimum sample of 35 patients will be recruited, allowing recruitment and retention rates to be estimated with 95% CI maximum widths of 2% and 28%.^30^

#### Data analysis

Simple descriptive statistics will be reported for each feasibility outcome measure (eg, number and proportion of eligible participants enrolled). All quantitative analyses will be conducted in SPSS or STATA. Interim analyses will be conducted 6 months after opening the study to recruitment.

Data will be collected from Atom5™ to assess the feasibility and acceptability of the PRO-CAR-T digital platform and *a priori* benchmarks applied:

- Retention: Number and proportion of patients who complete the 12-month PRO assessment. The study will be considered feasible if ≥ 70% of patients complete the final assessment at month 12.
- Adherence: Number and proportion of patients completing the PRO assessments. The study will be considered feasible if ≥ 70% of expected PRO assessments are completed at each time point, taking into consideration attrition arising from disease progression, severe toxicity resulting in loss of capacity or death. We will document reasons for non-completion (eg, hospital admission). Missed completions will be checked against the patient’s medical notes by a member of the clinical team (ie, research nurse) to identify possible reasons for non-completion (ie, a record of ICANS or other severe adverse treatment-related events, hospital admission or death). To be considered adherent, patients will need to have submitted their report via the PRO-CAR-T system within 72 hours of the scheduled time point. *Ad hoc* reports will not contribute to the assessment of adherence. Incomplete submissions will be accepted, although missing data are expected to be minimal with the required fields functionality in Atom5.
- Recruitment: Number and proportion (%) of eligible patients who consent to take part. The study will be considered feasible if ≥ 50% of eligible patients consent.

- Number of clinical alerts and number and proportion of patients reporting clinical alerts.
- Number and proportion of patients who withdraw formally from the study and their reasons for withdrawal.
- Actions arising from alerts including number and proportion of patients attending clinic, hospitalisation following clinical alert reporting, estimated time between alert and response by clinical team.
- Number of *ad hoc* PRO assessments completed and the number and proportion of patients submitting ad hoc PRO assessments.

At 3, 6 and 12 months postinfusion, acceptability will be assessed using implementation outcome measures: Acceptability of Intervention Measure (AIM), Intervention Appropriateness Measure (IAM) and Feasibility of Intervention Measure (FIM) scales. Psychometric evaluation showed acceptable internal consistency reliability (Cronbach’s alpha values ranging from 0.85 to 0.91) and test–retest reliability (Pearson correlation values ranging from 0.73 to 0.88), and a Flesch 5th reading grade level score. Each scale has four items and completion time for all three scales is less than 5 min. Participants will rate their agreement on a Likert scale in response to statements about using the PRO-CAR-T digital system (eg, ‘The PRO-CAR-T system is appealing to me.’).^31^

#### Qualitative substudy

We will conduct a qualitative evaluation of the PRO-CAR-T system with clinicians and a subsample of feasibility study participants at approximately 3 months postinfusion. This time interval was selected to ensure patients have sufficient opportunity to use the PRO-CAR-T system. Patients and HCPs who give their consent to participate will take part in an interview lasting up to 1 hour, with breaks as required. Semistructured topic guides (tailored to the different participant groups) will be used to ensure key topics are consistently covered. The topic guides will remain flexible and evolve based on findings. We will use reflexive thematic analysis for data analysis in NVivo with two researchers coding a subset of the transcripts to cross check the coding strategy and data interpretation.

## Patient and public involvement

The patient and public involvement and engagement (PPIE) plan for the PRO-CAR-T study sits within the overall Blood and Transplant Research Unit (BTRU) PPIE strategy.^32^ The strategy has been developed by patients, carers, members of the public and researchers who have worked together to agree a PPIE action plan informed by the UK Standards of Public Involvement in Research.^33^ In accordance with principles of coproduction and the BTRU PPIE Strategy, researchers will work in partnership with patient and public contributors through a mix of consultation, collaboration and user-led activities.^32^ Patient authors have been involved in the codesign of the research protocol, patient and public-facing materials such as lay summaries and patient information leaflets. Future activities will include supporting the interpretation of findings, codesigning engagement strategies and materials to share findings with the public (eg, lay summaries, press releases, creative and social media communications), coauthoring academic/clinical outputs and supporting research governance activities (eg, patients and public contributors are included in the BTRU Project Management Group).

## Ethics and dissemination

Ethical approval for the study was provided by the UK’s Health Research Authority Social Care Research Ethics Committee B Proportionate Review Subcommittee (HSC REC B) (ref: 23/NI/0104). The results of the study will be published in a peer-reviewed journal, presented at conferences and symposia and shared with patients and members of the public through lay summaries via multiple media.

## Supplementary Material

Reviewer comments

Author's manuscript
