# Supplementary material for: Protocol for a mixed-methods study to develop and feasibility test a digital system for the capture of patient-reported outcomes (PROs) in patients receiving chimeric antigen receptor T-cell (CAR-T) therapies (the PRO-CAR-T study)
Source: BMJ Open. 2024 Mar 29;14(3):e085392. doi: 10.1136/bmjopen-2024-085392 (PMC10982800; doi:10.1136/bmjopen-2024-085392)
Supplement: Author's manuscript [file bmjopen-2024-085392.draft_revisions.pdf]

BMJ Open

BMJ Open is committed to open peer review. As part of this commitment we make the peer review history of every article we publish publicly available.

When an article is published we post the peer reviewers' comments and the authors' responses online. We also post the versions of the paper that were used during peer review. These are the versions that the peer review comments apply to.

The versions of the paper that follow are the versions that were submitted during the peer review process. They are not the versions of record or the final published versions. They should not be cited or distributed as the published version of this manuscript.

BMJ Open is an open access journal and the full, final, typeset and author-corrected version of record of the manuscript is available on our site with no access controls, subscription charges or pay-per-view fees (http://bmjopen.bmj.com).

If you have any questions on BMJ Open's open peer review process please email info.bmjopen@bmj.com

BMJ Open

Protocol for a mixed-methods study to develop a digital solution for the capture of Patient-Reported Outcomes in patients receiving CAR T-cell therapies (The PRO-CAR-T Study)

Journal:	*BMJ Open*
Manuscript ID	bmjopen-2024-085392
Article Type:	Protocol
Date Submitted by the Author:	14-Feb-2024

Complete List of Authors:	Hughes, Sarah; University of Birmingham, Centre for Patient Reported Outcome Research, Institute of Applied Health Research<br>McMullan, Christel; University of Birmingham, Centre for Patient Reported Outcome Research, Institute of Applied Health Research<br>Aiyegbusi, Olalekan Lee; University of Birmingham, Centre for Patient Reported Outcome Research, Institute of Applied Health Research<br>Shaw, Karen; University of Birmingham, Centre for Patient Reported Outcome Research, Institute of Applied Health Research<br>Kinsella, Francesca; University Hospitals Birmingham NHS Foundation Trust; University of Birmingham<br>Ferguson, Paul; University Hospitals Birmingham NHS Foundation Trust<br>Khatsuria, Foram; University of Birmingham, Centre for Patient Reported Outcome Research, Institute of Applied Health Research<br>Burns, David; University Hospitals Birmingham NHS Foundation Trust<br>Pyatt, Lester; Patient partner<br>Ansell, John; Patient partner<br>Chakera, Evelyn; Patient partner<br>Richardson-Abraham, Julie; Patient partner<br>Denniston, Alastair; University Hospitals Birmingham NHS Foundation Trust; University of Birmingham<br>Davies, Elin Haf; Aparito Ltd<br>Craddock, Charles; University Hospitals Birmingham NHS Foundation Trust, Centre for Clinical Haematology; University of Birmingham<br>Calvert , Melanie ; University of Birmingham, Centre for Patient Reported Outcomes Research and Institute of Applied Health Research

Keywords:	Quality of Life, Patient-Centered Care, Gene therapy < ONCOLOGY, Patient Reported Outcome Measures, HAEMATOLOGY, eHealth

SCHOLARONE™
Manuscripts

Word count: 3303 (excluding abstract, figures, acknowledgments, references)

Protocol for a mixed-methods study to develop a digital solution for the capture of Patient-Reported Outcomes in patients receiving CAR T-cell therapies (The PRO-CAR-T Study)

Sarah E Hughes,[1-5] Christel McMullan,[1,2,5] Olalekan L Aiyegbusi,[1-5] Karen L Shaw,[1,2] Alastair K Denniston,[2,4,5,8] Francesca Kinsella,[7] Paul Ferguson,[7] Foram Khasturia,[1,2,5] Elin Haf Davies,[6] David Burns,[7] Lester Pyatt,[9] John Ansell,[9] Evelyn Chakera,[9] Julie Richardson-Abraham,[9] Charles Craddock,[7] Melanie Calvert[1-5]

[1]Centre for Patient-Reported Outcome Research (CPROR), Institute of Applied Health, University of Birmingham, Birmingham, UK
[2]National Institute of Health and Care Research (NIHR) Blood and Transplant Research Unit (BTRU) in Precision Cellular Therapeutics at the University of Birmingham, UK
[3]NIHR Applied Research Collaboration (ARC) West Midlands, Birmingham, UK
[4]Birmingham Health Partners Centre for Regulatory Science and Innovation, University of Birmingham, Birmingham, UK
[5]NIHR Birmingham Biomedical Research Centre, Birmingham, UK
[6]Aparito Limited
[7]Birmingham Centre for Cellular Therapy and Transplant, University Hospitals Birmingham NHS Trust, Birmingham, UK
[8]Academic Unit of Ophthalmology, Institute of Inflammation and Ageing, University of Birmingham, Birmingham, UK
[9]Patient Partner, Birmingham, UK

Address for correspondence:
Sarah Hughes
Centre for Patient Reported Outcome Research
Institute of Applied Health Research
Murray Learning Centre
University of Birmingham
Edgbaston
B15 2TT
e-mail: s.e.hughes@bham.ac.uk

Key words: PRO, patient-reported outcome, ePRO, electronic patient-reported outcome, chimeric antigen receptor T-cell, CAR-T, CAR T-cell, cellular therapy, advanced therapies, cancer, digital health, remote monitoring, symptom burden, neurotoxicity, cytokine release syndrome, ICANS, quality of life

For peer review only

ABSTRACT

Introduction: Chimeric Antigen Receptor (CAR) T-cell therapies are novel, potentially curative therapies for haematologic malignancies. CAR T-cell therapies are associated with severe toxicities, meaning patients require monitoring during acute and post-acute treatment phases. Electronic patient-reported outcomes (ePROs), self-reports of health status provided via online questionnaires, can complement clinician observation with potential to improve patient outcomes. This study will develop and evaluate feasibility of a new ePRO system for CAR-T patients in routine care.

Methods and analysis: Multi-phase, mixed-methods study involving multiple stakeholder groups (patients, family members, carers, clinicians, academics/researchers, policymakers). The intervention development phase comprises a Delphi study to select patient-reported outcome (PRO) measures for the digital system, a co-design workshop, and consensus meetings to establish thresholds for notifications to the clinical team if a patient reports severe symptoms or side effects. Usability testing will evaluate how users interact with the digital system and, lastly, we will evaluate ePRO system feasibility with 30 CAR-T patients (adults aged 18+ years) when used in addition to usual care. Feasibility study participants will use the ePRO system to submit self-reports of symptoms, treatment tolerability, and quality of life at specific time points. The CAR-T clinical team will respond to system notifications triggered by patients' submitted responses with actions in line with standard clinical practice. Feasibility measures will be collected at pre-specified timepoints following CAR T-cell infusion. A qualitative sub-study involving patients and clinical team members will explore acceptability of the ePRO system.

Ethics and dissemination: Favourable ethical opinion was granted by the Health and Social Care Research Ethics Committee B(HSC REC B) (Ref: 23/NI/0104) on 28th September 2023. Findings will be submitted for publication in high quality, peer-reviewed journals. Summaries of results, co-developed with the BTRU PPIE group, will be disseminated to all interested groups.

Trial Registration Number: ISCTRN Registration 11232653 (https://doi.org/10.1186/ISRCTN11232653).

For peer review only

For peer review only - http://bmjopen.bmj.com/site/about/guidelines.xhtml

STRENGTHS AND LIMITATIONS OF THIS STUDY

- The research site University Hospitals Birmingham NHS Foundation Trust hosts one of the largest cellular therapy centres in the United Kingdom.
- The planned study will evaluate the feasibility of the PRO-CAR-T system in the target population of patients with haematological malignancies who are recipients of CAR T-cell therapy. A fully powered randomised controlled trial will be required subsequently to establish the effectiveness of capturing patient-reported data using a digital system on clinical care and outcomes compared to standard practice in a real-world clinical setting.
- The study protocol was developed by a multi-disciplinary team of experts including patients and members of the public, healthcare professionals and academics.
- The PRO-CAR-T system will enable collection of longitudinal PRO data for up to 12-months post-infusion. Few studies have explored symptom burden and health-related quality of life (HRQoL) in CAR-T patients beyond the post-acute phase of treatment.

For peer review only - http://bmjopen.bmj.com/site/about/guidelines.xhtml

INTRODUCTION

Chimeric antigen receptor (CAR) T-cell therapies are innovative therapies for treatment of haematologic malignancies. Using T-cells that have been genetically engineered to redirect these cells' cytotoxic specificity towards tumour cells, CAR T-cell therapy has shown promising results in terms of the durability of remission, with a recent meta-analysis reporting a complete response in 54.4% of patients who received CD19 CAR T-cells across 27 studies.[1,2] However, these encouraging findings are tempered with significant safety concerns arising from unique treatment-related toxicities that can be life-threatening.[3]

Cytokine release syndrome (CRS) and Immune Effector Cell-associated Neurotoxicity Syndrome (ICANS) are the most commonly occurring toxicities following CAR-T-cell infusion. CRS is a systemic inflammatory response caused by cytokines released by the infused T-cells. Symptoms are numerous and heterogenous, affecting multiple organ systems and, in severe cases, can lead to organ failure.[4,5] CRS of any grade is estimated to present in a significant proportion of CAR-T patients with estimates ranging from 37-93% in patients with lymphoma and 77-93% of leukaemia patients.[3] ICANS can occur concurrently or after CRS. Signs and symptoms can include confusion, tremor, expressive aphasia, and seizures.[2,5,6]

Due to the unique toxicity profile of CAR-T cell therapies, patients receiving these novel treatments require intensive monitoring by their clinical team. Patient-reported outcomes (PROs) are reports of a person's health (i.e., symptoms, functioning, quality of life) that come directly from the individual without interpretation by a clinician or anyone else.[7] PROs can prove a useful complement to clinician observation. For example, a randomised controlled trial (RCT) of cancer patients receiving chemotherapy found that remote monitoring using PROs improved survival, reduced hospital admissions, and improved patients' quality of life compared to usual care.[8] Furthermore, evidence suggests clinicians may under-report the incidence and severity of adverse event-related toxicities. A systematic review found poor-to-moderate associations between analogous clinician-reported Common Terminology Criteria for Adverse Events and PROs, regardless of the PRO measure used.[9] PROs may also be useful in clinical practice to inform decision-making around treatment and care, support communication between the patient and their healthcare team, and aid self-management.[10,11] Whilst PRO data can help to ensure that patients' views about their treatment and side effects remain at the forefront of care, they are currently under-reported in clinical trials of CAR-T products and there is limited literature exploring the use of PROs for monitoring of CAR-T-related adverse events and quality of life in clinical settings, particularly in a UK context.[12]

The field of precision cellular therapy is forecast to undergo rapid expansion in the coming decade and there is an urgent need for evidence-based tools, co-produced with patients, to better support the growing number of individuals with haematologic malignancies who will receive these potentially curative treatments. The novelty of CAR-T cell therapies and the unique, potentially severe toxicities associated with these treatments mean effective symptom monitoring is key to maximising treatment benefit, ensuring patient safety, and reducing care costs. Remote monitoring using ePRO systems

For peer review only - http://bmjopen.bmj.com/site/about/guidelines.xhtml

could enable patients to be supported at home or otherwise away from the clinic and hospital ward. Findings from a recent pilot study have suggested that longitudinal PRO data capture is both feasible and acceptable for use with CAR-T cell therapy patients.[6]

The PRO-CAR-T Study aims to develop and assess the feasibility of an ePRO system for patients receiving CAR-T cell therapies and is aligned with research priorities highlighted in the UK Stem Cell Strategic Forum's report "A ten-year vision for stem cell transplantation and cellular therapies".[13] Development work will include concept elicitation and development of the conceptual framework and measurement strategy that will underpin the PRO-CAR-T system and co-design of the system including the format of alert notifications to the clinical team (when symptoms of concern are reported by the patient). We will evaluate the feasibility of the digital system with CAR-T therapy patients in a clinical setting to help inform the design of a full-scale RCT evaluating the effectiveness of ePRO remote monitoring in patients receiving CAR T-cell therapies.

AIMS AND OBJECTIVES

This mixed-methods multi-phase study aims to develop a new digital platform to capture PROs for remote monitoring of symptoms, side effects and health-related quality of life in patients with haematological malignancies receiving CAR T-cell therapies and assess its feasibility for use in the UK's National Health Service (NHS).

The key objectives are:

1. To develop a conceptual framework for measurement that will underpin the PRO-CAR-T digital system.
2. To identify and shortlist candidate patient-reported outcome (PRO) measures and map these to the digital system's conceptual framework for measurement.
3. To select, through a consensus-building process with stakeholders, the PRO measures for inclusion in the digital platform.
4. To identify items in the included PRO measures that assess symptoms and other constructs of clinical relevance or concern (e.g., severe side effects, treatment tolerability, quality of life) that require notifications to the clinical team through system alerts.
5. To understand stakeholder needs in relation to PRO-based alert management within routine CAR-T care and to co-develop the alert functionality for the digital platform.
6. To test the usability of the new digital system to ensure the platform is effective, efficient and perceived as satisfactory by end users.
7. To assess feasibility and acceptability of the new digital system to capture PROs within a routine CAR-T clinical setting.

METHODS AND ANALYSIS

Design

For peer review only

The PRO-CAR-T Study comprises three, sequential work packages or phases, each building upon the former. Work Package 1 (WP1) involves development of the digital intervention (ePRO system); Work Package 2 (WP2) involves building and testing system usability; and Work Package 3 (WP3) involves evaluating the feasibility of the digital system when it is deployed in a clinical setting and includes a qualitative sub-study (Fig 1).

[insert figure 1 here]

The Aparito Atom5™ platform

The PRO-CAR-T ePRO system will be deployed using the Atom5™ digital platform developed by Aparito Limited. Atom5™ is a configurable, multi-lingual, disease-agnostic digital platform for remote patient monitoring. It has several capabilities that make it ideally suited to patient monitoring following CAR-T cell therapy, including the capability to track symptoms and quality of life with the use of ePROs, deliver tailored notifications and alerts with links to relevant support services, and convey data in real-time to healthcare professionals and researchers. The platform comprises a patient-facing desktop portal accessed via an internet browser, patient-facing app (Android and iOS compatible), clinician/study staff-facing desktop web portal, and data analytics dashboard. Each deployment is GxP compliant. Aparito is accredited for Quality Management System (QMS), ISO 13485 and Information Security Management System (ISMS) ISO 27001.

Population and setting

CAR-T patients in the United Kingdom who are managed by the Birmingham Centre for Cellular Therapy and Transplant at Queen Elizabeth Hospital, University Hospitals Birmingham NHS Foundation Trust will be invited to participate in this study. Depending on the specific work package, other participant groups will include family members, healthcare professionals, academics/researchers, regulators, policymakers, and industry representatives.

Work Package 1: ePRO system development

The PRO-CAR-T digital system specification will be co-produced through a series of workshops and/or individual interviews (depending on participant preference) involving patients and their family members, healthcare professionals (HCPs), academics/researchers, policymakers, and industry representatives. First, a conceptual framework for measurement and shortlist of candidate PRO measures will be generated from a systematic search and rapid review of the published literature.[14] The conceptual framework will provide a content map describing the expected relationships between constructs to be measured by the PRO-CAR-T system and the items/domains of a PRO measure.[15] Next, a Delphi review panel involving up to 30 participants will select PRO measures for inclusion in the PRO-CAR-T system from the list of shortlisted measures. The Delphi study will comprise two rounds: 1) an online survey followed by 2) an online workshop to reach consensus on the PRO measures to be included.

For peer review only - http://bmjopen.bmj.com/site/about/guidelines.xhtml

Last, we will hold a series of workshops (or individual interviews depending on participant preference) to: 1) ascertain the essential and desirable components of an ePRO system (co-design) and 2) establish those items from the included PRO measures that, if reported by a CAR-T patient, would be of sufficient clinical concern to require notification to the patient's clinical team. We will explore co-design elements with participants including key stakeholders' requirements for symptom reporting, information, and communication mapped against the patient journey. Findings will ensure the design of the PRO-CAR-T system is grounded in the needs and expectations of users.[16] The co-design approach is based on ISO standards for human-centred design for interactive systems (European Committee for Standardisation 2010), ISPOR guidance on ePRO systems, and the user innovation management method.[16–18] To establish alerting requirements, workshops and interviews will explore stakeholders preferences for the format of alert notifications, establish scoring thresholds and arising actions, and map the end-to-end clinical workflow that will occur within the Atom5™ environment. We will invite up to 30 individuals to take part from the following participant groups: CAR-T patients and their family members, healthcare professionals and academics/researchers.

For each component of intervention development (Work Package 1), we will use descriptive statistics to analyse quantitative survey data (overall and per participant group) using Excel, STATA or SPSS. For qualitative analyses, we will apply thematic analysis to inductively code free text survey responses and workshop transcripts.[19] Two researchers will independently code a subset of the transcripts to cross check the coding strategy and data interpretation and the COREQ checklist will be used as a guideline for reporting qualitative methods and findings.[20] Deductive frameworks will support coding of data to develop the system specification and *a priori* criteria, based on Murphy et al. and Williamson et al. , will be applied to establish consensus on the PRO measures to be included in the PRO-CAR-T digital system.[21,22]

Work Package 2: Usability testing

Usability testing refers to the “formal assessment of the extent to which interaction with a product or system is effective, efficient and perceived as satisfactory by users” (p. 326)[23] and typically involves observation of end users interacting with the digital tool as they complete a series of tasks. CAR T-cell therapy patients and HCPs working in clinical settings offering CAR T-cell therapies will participate in a cognitive interview to evaluate the usability of the PRO-CAR-T system. A sample of 20-30 patients and HCPs will take part in two cycles of testing. The PRO-CAR-T system in Atom5™ will be refined iteratively after each test cycle until no new problems are encountered by participants. The usability test sessions will be conducted remotely using videoconferencing software. Test sessions will be recorded and transcribed verbatim for analysis. During the test session, patient participants will be asked to download Atom5™, access the PRO-CAR-T system, and navigate the system to complete the PRO measures. Whilst navigating the system, patients will be asked to “think aloud” to describe their thought process to the researcher. Patients will be informed that they do not have to answer the

For peer review only - http://bmjopen.bmj.com/site/about/guidelines.xhtml

questions about their symptoms or quality of life truthfully, that the emphasis of testing is to evaluate the PRO-CAR-T system's ease of use from the user perspective, and that any data they enter will not be saved nor used to inform their care. HCPs will login to the PRO-CAR-T clinical dashboard and complete specific tasks (e.g., register a patient, respond to a clinical alert) whilst thinking aloud. In the case of HCPs, fictitious patient data will be used. Upon completing the tasks, participants will be asked to provide feedback relating to their experience using the digital system including ways the user experience could be improved.

A semi-structured topic guide will be used to structure the interview and participants will also complete the System Usability Scale (SUS), a 10-item questionnaire providing an assessment of usability.[24] SUS items are scored on a 5-point rating scale ranging from "strongly disagree" to "strongly agree". Raw scores are converted to 0-100 score with higher scores indicating greater usability. A score greater than 80 is considered evidence of above average user experience.[25] The psychometric properties of the SUS have been evaluated extensively and the scale has been shown to have good internal consistency reliability (Cronbach's alpha = 0.83-0.91) and concurrent validity with other measures of usability.[26] A multi-method approach to usability (i.e., usability metrics, self-completed surveys, and cognitive interviewing techniques) was selected to counter positive recall bias associated with think aloud techniques.[23]

Data analysis will be guided by the Fit Between Individuals, Task and Technology (FITT) framework to describe usability on three dimensions: 1) task-technology fit; 2) individual-technology fit; and 3) individual-task fit.[27] Developed specifically for use in healthcare, the FITT framework enables developers to understand the relationship between users, tasks and technology and the factors affecting optimal device use.[28] Descriptive statistics will be used to describe the demographic characteristics of the study sample, SUS scores, prompt/error rates, and task completion times. Excel and SPSS or STATA statistics software will be used for all statistical analyses.

Cognitive "think aloud" interviews will be qualitatively analysed using thematic analysis and the framework method.[29] A deductive coding framework will be used to code data according to the FITT model and inductive coding will identify additional concepts in the data. NVivo (Version 14) qualitative data analysis software will be used to manage and code the transcribed interview data. Two researchers will independently code a subset of the data to cross check the coding strategy and data interpretation. Problems with the ePRO system identified during usability testing will be logged, discussed with Aparito, and a system change request generated. Refinements to the system will be made before commencing the next test cycle.

Work Package 3: Feasibility study

The PRO-CAR-T feasibility study is a single centre study of the feasibility and acceptability of the new ePRO system when deployed in the UK's National Health Service (NHS). Thirty CAR-T patients will be recruited consecutively to use the PRO-CAR-T system (i.e., in addition to usual care). Patients who consent to participate will be onboarded to the system prior to infusion. During the baseline visit, participants will download the Atom5™ app to their mobile device, give their demographic details

For peer review only - http://bmjopen.bmj.com/site/about/guidelines.xhtml

including age, sex at birth, ethnicity, cancer diagnosis, CAR T-cell therapy, and self-reported experience with technology and complete the PRO measures for the first time. PRO measures will be completed subsequently via the PRO-CAR-T system at pre-specified intervals (measures and frequency of assessments will be established in Work Package 1). We will follow participants for 12-months from date of infusion (Day 0) to understand the feasibility and acceptability of longitudinal collection of PROs for CAR-T patients, their families, and HCPs. The findings will be used to inform a future, definitive randomised controlled trial to assess the effectiveness of remote symptom monitoring using the digital system alongside usual care compared to usual care alone.

Sample size justification

There is no definitive guidance relating to sample sizes in pilot and feasibility studies with a heuristic of 30 (range = 10 to 40) patients generally applied. Therefore, no formal sample size calculation has been performed for the feasibility study. To allow for a 15% attrition rate, a minimum sample of 35 patients will be recruited, allowing recruitment and retention rates to be estimated with 95% confidence interval (CI) maximum widths of 2% and 28%.[30]

Data analysis

Simple descriptive statistics will be reported for each feasibility outcome measure (e.g., number and proportion of eligible participants enrolled). All quantitative analyses will be conducted in SPSS or STATA. Interim analyses will be conducted 6-months after opening the study to recruitment.

Data will be collected from Atom5™ to assess the feasibility and acceptability of the PRO-CAR-T digital platform and *a priori* benchmarks applied:

- *Recruitment*: Number and proportion (%) of eligible patients who consent to take part. The study will be considered feasible if $\geq$ 50% of eligible patients consent.
- *Retention*: Number and proportion of patients who complete the 12-month PRO assessment. The study will be considered feasible if $\geq$ 70% of patients complete the final assessment at Month-12.
- *Adherence*: Number and proportion of patients completing the PRO assessments. The study will be considered feasible if $\geq$ 70% of expected PRO assessments are completed at each timepoint, taking into consideration attrition arising from disease progression, severe toxicity resulting in loss of capacity, or death. We will document reasons for non-completion (e.g., hospital admission). Missed completions will be checked against the patient's medical notes by a member of the clinical team (i.e., research nurse) to identify possible reasons for non-completion (i.e., a record of ICANS or other severe adverse treatment-related events, hospital admission, or death). To be considered adherent, patients will need to have submitted their report via the PRO-CAR-T system within 72 hours of the scheduled timepoint. *Ad hoc* reports will not contribute to the assessment of adherence. Incomplete submissions will be accepted, although missing data is expected to be minimal with the required fields functionality in Atom5™.

For peer review only - http://bmjopen.bmj.com/site/about/guidelines.xhtml

- Number of clinical alerts and number and proportion of patients reporting clinical alerts.
- Number and proportion of patients who withdraw formally from the study and their reasons for withdrawal.
- Actions arising from alerts including number and proportion of patients attending clinic, hospitalisation following clinical alert reporting, estimated time between alert and response by clinical team.
- Number of *ad hoc* PRO assessments completed and the number and proportion of patients submitting *ad hoc* PRO assessments.

At 3-months, 6-months and 12-months post-infusion, acceptability will be assessed using implementation outcome measures: Acceptability of Intervention Measure (AIM), Intervention Appropriateness Measure (IAM), and Feasibility of Intervention Measure (FIM) scales. Psychometric evaluation showed acceptable internal consistency reliability (Cronbach's alpha values ranging from 0.85 – 0.91) and test-retest reliability (Pearson correlation values ranging from 0.73 – 0.88), and a Flesch 5th reading grade level score. Each scale has four items and completion time for all three scales is less than five minutes. Participants will rate their agreement on a Likert scale in response to statements about using the PRO-CAR-T digital system (e.g., "The PRO-CAR-T system is appealing to me.").[31]

Qualitative sub-study

We will conduct a qualitative evaluation of the PRO-CAR-T system with clinicians and a sub-sample of feasibility study participants at approximately 3-months post-infusion. This time interval was selected to ensure patients have sufficient opportunity to use the PRO-CAR-T system. Patients and HCPs who give their consent to participate will take part in an interview lasting up to one hour, with breaks as required. Semi-structured topic guides (tailored to the different participant groups) will be used to ensure key topics are consistently covered. The topic guides will remain flexible and evolve based on findings. We will use reflexive thematic analysis for data analysis in NVivo with two researchers coding a subset of the transcripts to cross check the coding strategy and data interpretation.

PATIENT AND PUBLIC INVOLVEMENT

The Patient and Public Involvement and Engagement (PPIE) plan for the PRO-CAR-T Study sits within the overall BTRU PPIE strategy.[32] The strategy has been developed by patients, carers, members of the public, and researchers who have worked together to agree a PPIE action-plan informed by the UK Standards of Public Involvement in Research.[33] In accordance with principles of co-production and the BTRU PPIE Strategy, researchers will work in partnership with patient and public contributors through a mix of consultation, collaboration and user-led activities.[32] Patient partners have been involved in the co-design of the research protocol, patient and public-facing materials such as lay summaries and patient information leaflets. Future activities will include supporting the interpretation of findings, co-designing engagement strategies and materials to share findings with the public (e.g., lay summaries, press releases, creative and social media communications), co-authoring

For peer review only

For peer review only - http://bmjopen.bmj.com/site/about/guidelines.xhtml

academic/clinical outputs, and supporting Research Governance activities (e.g., patients and public contributors are included in the BTRU Project Management Group).

ETHICS AND DISSEMINATION

Ethical approval for the study was provided by the UK's Health Research Authority (HRA) Social Care Research Ethics Committee B Proportionate Review Subcommittee (HSC REC B) (Ref: 23/NI/0104). The results of the study will be published in a peer-reviewed journal, presented at conferences and symposia and shared with patients and members of the public through lay summaries via multiple media.

For peer review only

ACKNOWLEDGMENTS

We thank the Blood and Transplant Research Unit in Precision Cellular Therapeutics Patient and Public Involvement (PPIE) Group for their help and support in developing the protocol for the PRO-CAR-T Study.

Contributors: MC, OLA, SEH, DB, CC, FrK, AD, EHD designed the study; SEH developed the first draft of the protocol. DB, PF, FrK, AD, CC provided clinical expertise; MC, SEH, CM, OLA, KS, FK, EDH provided methodological expertise; LP, JA, EC, JRA and the BTRU PPIE group provided patient input. All investigators provided advice and critical input to the study design. All authors provided critical review of the manuscript and approved the final version.

Funding: This study is sponsored by the University of Birmingham and funded by the National Institute for Health and Care Research (NIHR) Blood and Transplant Research Unit (BTRU) in Precision Cellular Therapeutics (Reference: NIHR 203339). The views expressed are those of the author(s) and not necessarily those of the NIHR or the Department of Health and Social Care.

Competing interests: MC is Director of the Birmingham Health Partners Centre for Regulatory Science and Innovation, Director of the Centre for the Centre for Patient Reported Outcomes Research and is a National Institute for Health Research (NIHR) Senior Investigator. MC receives funding from the National Institute for Health Research (NIHR), UK Research and Innovation (UKRI), NIHR Birmingham Biomedical Research Centre, the NIHR Surgical Reconstruction and Microbiology Research Centre, NIHR ARC West Midlands, UK SPINE, European Regional Development Fund – Demand Hub and Health Data Research UK at the University of Birmingham and University Hospitals Birmingham NHS Foundation Trust, Innovate UK (part of UKRI), Macmillan Cancer Support, UCB Pharma, Anthony Nolan, GSK and Gilead. MJC has received personal fees from Astellas, Aparito Ltd, CIS Oncology, Takeda, Merck, Daiichi Sankyo, Glaukos, GSK and the Patient-Centered Outcomes Research Institute (PCORI) outside the submitted work. In addition, a family member owns shares in GSK. **OLA** receives funding from the NIHR Birmingham BRC, West Midlands, Birmingham, NIHR ARC West Midlands, NIHR BTRU, UKRI, Health Foundation, Anthony Nolan, Gilead Sciences Ltd, Sarcoma UK, and GSK. He declares personal fees from Gilead Sciences Ltd, Merck and GSK outside the submitted work. **SEH** receives funding from the NIHR, UKRI, Rinri Therapeutics, Anthony Nolan, and declares personal fees from Cochlear Ltd, Astra Zeneca, Pfizer, CIS Oncology, and Aparito Ltd outside the submitted work. **CM** receives funding from NIHR Surgical Reconstruction and Microbiology Research Centre (SRMRC), UKRI, Anthony Nolan, and declares personal fees from Aparito Ltd outside the submitted work. All other authors declare no competing interests.

Patient consent for publication: Not required.

Provenance and peer review: Not commissioned; peer reviewed for ethical and funding approval prior to submission.

Open access: This is an open access article distributed in accordance with the Creative Commons Attribution 4.0 International (CC BY 4.0) licence, which permits others to copy, redistribute, remix,

For peer review only - http://bmjopen.bmj.com/site/about/guidelines.xhtml

transform and build upon this work for any purpose, provided the original work is properly cited, a link to the licence is given, and indication of whether changes were made. See: https://creativecommons.org/ licenses/by/4.0/.

ORCiDs

Sarah E Hughes: 0000-0001-5656-1198

Olalekan L Aiyegbusi: 0000-0001-9122-8251

Christel McMullan: 0000-0002-0878-1513

Karen Shaw: 0000-0001-7120-8595

Alastair Denniston: 0000-0001-7849-0087

Francesca Kinsella: 0000-0002-6022-2567

Foram Khasturia: 0009-0004-3076-0593

Elin Haf Davies: 0000-0002-4987-0088

Charles Craddock: 0000-0001-5041-6678

Melanie Calvert 0000-0002-1856-837X

For peer review only

REFERENCES

1 Grigor EJM, Fergusson D, Kekre N, *et al.* Risks and Benefits of Chimeric Antigen Receptor T-Cell (CAR-T) Therapy in Cancer: A Systematic Review and Meta-Analysis. *Transfus Med Rev*. 2019;33:98–110.

2 Neelapu SS, Tummala S, Kebriaei P, *et al.* Toxicity management after chimeric antigen receptor T cell therapy: one size does not fit "ALL." *Nat Rev Clin Oncol*. 2018;15:218.

3 Santomasso B, Bachier C, Westin J, *et al.* The Other Side of CAR T-Cell Therapy: Cytokine Release Syndrome, Neurologic Toxicity, and Financial Burden. *American Society of Clinical Oncology Educational Book*. 2019;433–44.

4 Whisenant MS, Srour SA, Williams LA, *et al.* The Unique Symptom Burden of Patients Receiving CAR T-Cell Therapy. *Semin Oncol Nurs*. 2021;37:151216.

5 Brudno JN, Kochenderfer JN. Toxicities of chimeric antigen receptor T cells: recognition and management. *Blood*. 2016;127:3321–30.

6 Oswald LB, Li X, Carvajal R, *et al.* Longitudinal Collection of Patient-Reported Outcomes and Activity Data during CAR-T Therapy: Feasibility, Acceptability, and Data Visualization. *Cancers*. 2022;14:2742. doi: 10.3390/cancers14112742

7 US Food and Drug Administration. Principles for Selecting, Developing, Modifying, and Adapting Patient-Reported Outcome Instruments for Use in Medical Device Evaluation: Draft Guidance for Industry and Food and Drug Administration Staff, And Other Stakeholders. 2020.

8 Basch E, Deal AM, Kris MG, *et al.* Symptom monitoring with patient-reported outcomes during routine cancer treatment: A randomized controlled trial. *Journal of Clinical Oncology*. 2016;34:557–65.

9 Atkinson TM, Ryan SJ, Bennett AV, *et al.* The Association between Clinician-Based Common Terminology Criteria for Adverse Events (CTCAE) and Patient-Reported Outcomes (PRO): A Systematic Review. *Support Care Cancer*. 2016;24:3669–76.

For peer review only - http://bmjopen.bmj.com/site/about/guidelines.xhtml

10 Calvert M, Kyte D, Price G, *et al.* Maximising the impact of patient reported outcome assessment for patients and society. *BMJ*. 2019;364. doi: 10.1136/bmj.k5267

11 Greenhalgh J, Gooding K, Gibbons E, *et al.* How do patient reported outcome measures (PROMs) support clinician-patient communication and patient care? A realist synthesis. *Journal of Patient-Reported Outcomes*. 2018;2. doi: 10.1186/s41687-018-0061-6

12 Efficace F, Cannella L, Sparano F, *et al.* Chimeric Antigen Receptor T-cell Therapy in Hematologic Malignancies and Patient-reported Outcomes: A Scoping Review. *HemaSphere*. 2022;6:e802.

13 UK Stem Cell Strategic Forum. A ten year vision for stem cell transplantation and cellular therapies. 2022.

14 Khatsuria F, Calvert M, McMullan C, *et al.* Protocol for a rapid review to inform concept elicitation and measure selection for an electronic patient-reported outcome (ePRO) system for patients with haematological malignancies receiving CAR-T cellular therapies. Published Online First: 12 May 2023. doi: 10.17605/OSF.IO/A6QBX

15 Donatti C, Wild D, Hareendran A. The use of conceptual models, conceptual frameworks, and endpoint models to support label claims of treatment benefit using patient reported outcomes. *ISPOR Connections*. ;9–12.

16 Nielsen AC, Rotger-Griful S, Kanstrup AM, *et al.* User-Innovated eHealth Solutions for Service Delivery to Older Persons With Hearing Impairment. *Am J Audiol*. 2018;27:403–16.

17 Zbrozek A, Hebert J, Gogates G, *et al.* Validation of Electronic Systems to Collect Patient-Reported Outcome (PRO) Data—Recommendations for Clinical Trial Teams: Report of the ISPOR ePRO Systems Validation Good Research Practices Task Force. *Value in Health*. 2013;16:480–9.

18 Kanstrup AM, Bertelsen PS. User Innovation Management: A Handbook. 2011.

19 Braun V, Clarke V. *Thematic analysis: a practical guide*. London: SAGE Publications 2022.

20 Tong A, Sainsbury P, Craig J. Consolidated criteria for reporting qualitative research (COREQ): a 32-item checklist for interviews and focus groups. *International Journal for Quality in Health Care*. 2007;19:349–57.

21 Murphy M, Hollinghurst S, Salisbury C. Agreeing the content of a patient-reported outcome measure for primary care: a Delphi consensus study. *Health Expectations*. 2017;20:335–48.

22 Williamson PR, Altman DG, Bagley H, *et al.* The COMET Handbook: version 1.0. *Trials*. 2017;18:280.

23 Aiyegbusi OL. Key methodological considerations for usability testing of electronic patient-reported outcome (ePRO) systems. *Qual Life Res*. 2020;29:325–33.

24 Brooke J. SUS: A quick and dirty usability scale. *Usability Eval Ind*. 1995;189.

25 Lewis JR, Sauro J. Item benchmarks for the system usability scale. *Journal of Usability Studies*. 2018;13.

26 Lewis JR. The System Usability Scale: Past, Present, and Future. *International Journal of Human–Computer Interaction*. 2018;34:577–90.

27 Ammenwerth E, Iller C, Mahler C. IT-adoption and the interaction of task, technology and individuals: a fit framework and a case study. *BMC Medical Informatics and Decision Making*. 2006;6:3.

28 Prgomet M, Georgiou A, Callen J, *et al.* Fit Between Individuals, Tasks, Technology, and Environment (FITTE) Framework: A Proposed Extension of FITT to Evaluate and Optimise Health Information Technology Use. *MEDINFO 2019: Health and Wellbeing e-Networks for All*. IOS Press 2019:744–8. https://doi.org/10.3233/SHTI190322

For peer review only - http://bmjopen.bmj.com/site/about/guidelines.xhtml

29 Gale NK, Heath G, Cameron E, *et al.* Using the framework method for the analysis of qualitative data in multi-disciplinary health research. *BMC Med Res Methodol*. 2013;13:117.

30 Herzog M. Considerations in Determining Sample Size for Pilot Studies. *Research in Nursing & Health*. 2008;31:180–91.

31 Weiner BJ, Lewis CC, Stanick C, *et al.* Psychometric assessment of three newly developed implementation outcome measures. *Implementation Science*. 2017;12:108.

32 NIHR BTRU in Precision Cellular Therapeutics. NIHR BTRU in Precision Cellular Therapeutics. Patient and Public Involvement and Engagement Strategy, 2022-2027. 2022.

33 UK Standards for Public Involvement. Better public involvement for better health and social care research. 2019. https://sites.google.com/nihr.ac.uk/pi-standards/home

For peer review only

For peer review only - http://bmjopen.bmj.com/site/about/guidelines.xhtml

For peer review only

For peer review only - http://bmjopen.bmj.com/site/about/guidelines.xhtml

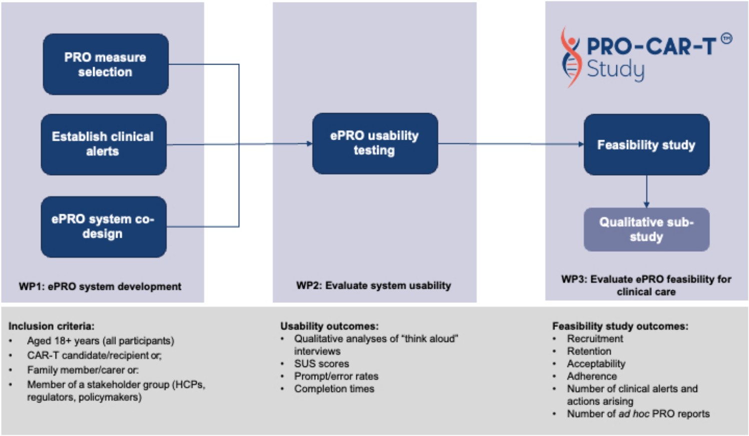

Figure 1. Study flow diagram for the PRO-CAR-T Study. SUS, System Usability Scale; HCP, Healthcare Professional; CAR-T, Chimeric Antigen Receptor T-cell; ePRO, electronic patient-reported outcome; PRO, patient-reported outcome.

338x190mm (72 x 72 DPI)

BMJ Open

Protocol for a mixed-methods study to develop and feasibility test a digital system for the capture of Patient-Reported Outcomes (PROs) in patients receiving Chimeric Antigen Receptor T-cell (CAR-T) therapies (The PRO-CAR-T Study)

Journal:	*BMJ Open*
Manuscript ID	bmjopen-2024-085392.R1
Article Type:	Protocol
Date Submitted by the Author:	14-Mar-2024

Complete List of Authors:	Hughes, Sarah; University of Birmingham, Centre for Patient Reported Outcome Research, Institute of Applied Health Research<br>McMullan, Christel; University of Birmingham, Centre for Patient Reported Outcome Research, Institute of Applied Health Research<br>Aiyegbusi, Olalekan Lee; University of Birmingham, Centre for Patient Reported Outcome Research, Institute of Applied Health Research<br>Shaw, Karen; University of Birmingham, Centre for Patient Reported Outcome Research, Institute of Applied Health Research<br>Kinsella, Francesca; University Hospitals Birmingham NHS Foundation Trust; University of Birmingham<br>Ferguson, Paul; University Hospitals Birmingham NHS Foundation Trust<br>Khatsuria, Foram; University of Birmingham, Centre for Patient Reported Outcome Research, Institute of Applied Health Research<br>Burns, David; University Hospitals Birmingham NHS Foundation Trust<br>Pyatt, Lester; Patient Author<br>Ansell, John; Patient Author<br>Chakera, Evelyn; Patient Author<br>Richardson-Abraham, Julie; Patient Author<br>Denniston, Alastair; University Hospitals Birmingham NHS Foundation Trust; University of Birmingham<br>Davies, Elin Haf; Aparito Ltd<br>Craddock, Charles; University Hospitals Birmingham NHS Foundation Trust, Centre for Clinical Haematology; University of Birmingham<br>Calvert , Melanie ; University of Birmingham, Centre for Patient Reported Outcomes Research and Institute of Applied Health Research

**Primary Subject Heading**:	Haematology (incl blood transfusion)
Secondary Subject Heading:	Patient-centred medicine, Oncology, Health services research
Keywords:	Quality of Life, Patient-Centered Care, Gene therapy < ONCOLOGY, Patient Reported Outcome Measures, HAEMATOLOGY, eHealth

Word count: 3303 (excluding abstract, figures, acknowledgments, references)

Protocol for a mixed-methods study to develop and feasibility test a digital system for the capture of Patient-Reported Outcomes (PROs) in patients receiving Chimeric Antigen Receptor T-cell (CAR-T) therapies (The PRO-CAR-T Study)

Sarah E Hughes,[1-5] Christel McMullan,[1,2,5] Olalekan L Aiyegbusi,[1-5] Karen L Shaw,[1,2] Alastair K Denniston,[2,4,5,8] Francesca Kinsella,[7] Paul Ferguson,[7] Foram Khatsuria,[1,2,5] Elin Haf Davies,[6] David Burns,[7] Lester Pyatt,[9] John Ansell,[9] Evelyn Chakera,[9] Julie Richardson-Abraham,[9] Charles Craddock,[7] Melanie Calvert[1-5]

[1]Centre for Patient-Reported Outcome Research (CPROR), Institute of Applied Health, University of Birmingham, Birmingham, UK
[2]National Institute of Health and Care Research (NIHR) Blood and Transplant Research Unit (BTRU) in Precision Cellular Therapeutics at the University of Birmingham, UK
[3]NIHR Applied Research Collaboration (ARC) West Midlands, Birmingham, UK
[4]Birmingham Health Partners Centre for Regulatory Science and Innovation, University of Birmingham, Birmingham, UK
[5]NIHR Birmingham Biomedical Research Centre, Birmingham, UK
[6]Aparito Limited
[7]Birmingham Centre for Cellular Therapy and Transplant, University Hospitals Birmingham NHS Trust, Birmingham, UK
[8]Academic Unit of Ophthalmology, Institute of Inflammation and Ageing, University of Birmingham, Birmingham, UK
[9]Patient Author, Birmingham, UK

Address for correspondence:
Sarah Hughes
Centre for Patient Reported Outcome Research
Institute of Applied Health Research
Murray Learning Centre
University of Birmingham
Edgbaston
B15 2TT
e-mail: s.e.hughes@bham.ac.uk

Key words: PRO, patient-reported outcome, ePRO, electronic patient-reported outcome, chimeric antigen receptor T-cell, CAR-T, CAR T-cell, cellular therapy, advanced therapies, cancer, digital health, remote monitoring, symptom burden, neurotoxicity, cytokine release syndrome, ICANS, quality of life

For peer review only - http://bmjopen.bmj.com/site/about/guidelines.xhtml

For peer review only

ABSTRACT

Introduction: Chimeric Antigen Receptor (CAR) T-cell therapies are novel, potentially curative therapies for haematologic malignancies. CAR T-cell therapies are associated with severe toxicities, meaning patients require monitoring during acute and post-acute treatment phases. Electronic patient-reported outcomes (ePROs), self-reports of health status provided via online questionnaires, can complement clinician observation with potential to improve patient outcomes. This study will develop and evaluate feasibility of a new ePRO system for CAR-T patients in routine care.

Methods and analysis: Multi-phase, mixed-methods study involving multiple stakeholder groups (patients, family members, carers, clinicians, academics/researchers, policymakers). The intervention development phase comprises a Delphi study to select patient-reported outcome (PRO) measures for the digital system, a co-design workshop, and consensus meetings to establish thresholds for notifications to the clinical team if a patient reports severe symptoms or side effects. Usability testing will evaluate how users interact with the digital system and, lastly, we will evaluate ePRO system feasibility with 30 CAR-T patients (adults aged 18+ years) when used in addition to usual care. Feasibility study participants will use the ePRO system to submit self-reports of symptoms, treatment tolerability, and quality of life at specific time points. The CAR-T clinical team will respond to system notifications triggered by patients' submitted responses with actions in line with standard clinical practice. Feasibility measures will be collected at pre-specified timepoints following CAR T-cell infusion. A qualitative sub-study involving patients and clinical team members will explore acceptability of the ePRO system.

Ethics and dissemination: Favourable ethical opinion was granted by the Health and Social Care Research Ethics Committee B(HSC REC B) (Ref: 23/NI/0104) on 28th September 2023. Findings will be submitted for publication in high quality, peer-reviewed journals. Summaries of results, co-developed with the BTRU PPIE group, will be disseminated to all interested groups.

Trial Registration Number: ISCTRN Registration 11232653 (https://doi.org/10.1186/ISRCTN11232653).

For peer review only - http://bmjopen.bmj.com/site/about/guidelines.xhtml

STRENGTHS AND LIMITATIONS OF THIS STUDY

- Data for the study are strengthened by inclusion of multiple stakeholder groups, including patients, carers, healthcare professionals, academics, and policy makers.
- Consensus methods will be deployed at all stages of intervention development, ensuring the views of relevant groups are incorporated into the system's design.
- Applying the Fit Between Individuals, Task and Technology (FITT) framework and the use of validated questionnaires will enable theory-driven interrogation of ePRO system usability.
- Semi-structured interviews in the qualitative sub-study will permit in-depth exploration of system feasibility and acceptability with patients and clinicians.
- Duration of feasibility study means monitoring of CAR-T patients beyond 12-months post-infusion will not be undertaken.

For peer review only

For peer review only - http://bmjopen.bmj.com/site/about/guidelines.xhtml

INTRODUCTION

Chimeric antigen receptor (CAR) T-cell therapies are innovative therapies for treatment of haematologic malignancies. Using T-cells that have been genetically engineered to redirect these cells' cytotoxic specificity towards tumour cells, CAR T-cell therapy has shown promising results in terms of the durability of remission, with a recent meta-analysis reporting a complete response in 54.4% of patients who received CD19 CAR T-cells across 27 studies.[1,2] However, these encouraging findings are tempered with significant safety concerns arising from unique treatment-related toxicities that can be life-threatening.[3]

Cytokine release syndrome (CRS) and Immune Effector Cell-associated Neurotoxicity Syndrome (ICANS) are the most commonly occurring toxicities following CAR-T-cell infusion. CRS is a systemic inflammatory response caused by cytokines released by the infused T-cells. Symptoms are numerous and heterogenous, affecting multiple organ systems and, in severe cases, can lead to organ failure.[4,5] CRS of any grade is estimated to present in a significant proportion of CAR-T patients with estimates ranging from 37-93% in patients with lymphoma and 77-93% of leukaemia patients.[3] ICANS can occur concurrently or after CRS. Signs and symptoms can include confusion, tremor, expressive aphasia, and seizures.[2,5,6]

Due to the unique toxicity profile of CAR-T cell therapies, patients receiving these novel treatments require intensive monitoring by their clinical team. Patient-reported outcomes (PROs) are reports of a person's health (i.e., symptoms, functioning, quality of life) that come directly from the individual without interpretation by a clinician or anyone else.[7] PROs can prove a useful complement to clinician observation. For example, a randomised controlled trial (RCT) of cancer patients receiving chemotherapy found that remote monitoring using PROs improved survival, reduced hospital admissions, and improved patients' quality of life compared to usual care.[8] Furthermore, evidence suggests clinicians may under-report the incidence and severity of adverse event-related toxicities. A systematic review found poor-to-moderate associations between analogous clinician-reported Common Terminology Criteria for Adverse Events and PROs, regardless of the PRO measure used.[9] PROs may also be useful in clinical practice to inform decision-making around treatment and care, support communication between the patient and their healthcare team, and aid self-management.[10,11] Whilst PRO data can help to ensure that patients' views about their treatment and side effects remain at the forefront of care, they are currently under-reported in clinical trials of CAR-T products and there is limited literature exploring the use of PROs for monitoring of CAR-T-related adverse events and quality of life in clinical settings, particularly in a UK context.[12]

The field of precision cellular therapy is forecast to undergo rapid expansion in the coming decade and there is an urgent need for evidence-based tools, co-produced with patients, to better support the growing number of individuals with haematologic malignancies who will receive these potentially curative treatments. The novelty of CAR-T cell therapies and the unique, potentially severe toxicities associated with these treatments mean effective symptom monitoring is key to maximising treatment benefit, ensuring patient safety, and reducing care costs. Remote monitoring using ePRO systems

For peer review only

For peer review only - http://bmjopen.bmj.com/site/about/guidelines.xhtml

could enable patients to be supported at home or otherwise away from the clinic and hospital ward. Findings from a recent pilot study have suggested that longitudinal PRO data capture is both feasible and acceptable for use with CAR-T cell therapy patients.[6]

The PRO-CAR-T Study aims to develop and assess the feasibility of an ePRO system for patients receiving CAR-T cell therapies and is aligned with research priorities highlighted in the UK Stem Cell Strategic Forum's report "A ten-year vision for stem cell transplantation and cellular therapies".[13] Development work will include concept elicitation and development of the conceptual framework and measurement strategy that will underpin the PRO-CAR-T system and co-design of the system including the format of alert notifications to the clinical team (when symptoms of concern are reported by the patient). We will evaluate the feasibility of the digital system with CAR-T therapy patients in a clinical setting to help inform the design of a full-scale RCT evaluating the effectiveness of ePRO remote monitoring in patients receiving CAR T-cell therapies.

AIMS AND OBJECTIVES

This mixed-methods multi-phase study aims to develop a new digital platform to capture PROs for remote monitoring of symptoms, side effects and health-related quality of life in patients with haematological malignancies receiving CAR T-cell therapies and assess its feasibility for use in the UK's National Health Service (NHS).

The key objectives are:

1. To develop a conceptual framework for measurement that will underpin the PRO-CAR-T digital system.
2. To identify and shortlist candidate patient-reported outcome (PRO) measures and map these to the digital system's conceptual framework for measurement.
3. To select, through a consensus-building process with stakeholders, the PRO measures for inclusion in the digital platform.
4. To identify items in the included PRO measures that assess symptoms and other constructs of clinical relevance or concern (e.g., severe side effects, treatment tolerability, quality of life) that require notifications to the clinical team through system alerts.
5. To understand stakeholder needs in relation to PRO-based alert management within routine CAR-T care and to co-develop the alert functionality for the digital platform.
6. To test the usability of the new digital system to ensure the platform is effective, efficient and perceived as satisfactory by end users.
7. To assess feasibility and acceptability of the new digital system to capture PROs within a routine CAR-T clinical setting.

METHODS AND ANALYSIS

Design

For peer review only - http://bmjopen.bmj.com/site/about/guidelines.xhtml

The PRO-CAR-T Study comprises three, sequential work packages or phases, each building upon the former. Work Package 1 (WP1) involves development of the digital intervention (ePRO system); Work Package 2 (WP2) involves building and testing system usability; and Work Package 3 (WP3) involves evaluating the feasibility of the digital system when it is deployed in a clinical setting and includes a qualitative sub-study (Fig 1).

[insert figure 1 here]

The Aparito Atom5™ platform

The PRO-CAR-T ePRO system will be deployed using the Atom5™ digital platform developed by Aparito Limited. Atom5™ is a configurable, multi-lingual, disease-agnostic digital platform for remote patient monitoring. It has several capabilities that make it ideally suited to patient monitoring following CAR-T cell therapy, including the capability to track symptoms and quality of life with the use of ePROs, deliver tailored notifications and alerts with links to relevant support services, and convey data in real-time to healthcare professionals and researchers. The platform comprises a patient-facing desktop portal accessed via an internet browser, patient-facing app (Android and iOS compatible), clinician/study staff-facing desktop web portal, and data analytics dashboard. Each deployment is GxP compliant. Aparito is accredited for Quality Management System (QMS), ISO 13485 and Information Security Management System (ISMS) ISO 27001.

Population and setting

CAR-T patients in the United Kingdom who are managed by the Birmingham Centre for Cellular Therapy and Transplant at Queen Elizabeth Hospital, University Hospitals Birmingham NHS Foundation Trust will be invited to participate in this study. Depending on the specific work package, other participant groups will include family members, healthcare professionals, academics/researchers, regulators, policymakers, and industry representatives.

Work Package 1: ePRO system development

The PRO-CAR-T digital system specification will be co-produced through a series of workshops and/or individual interviews (depending on participant preference) involving patients and their family members, healthcare professionals (HCPs), academics/researchers, policymakers, and industry representatives. First, a conceptual framework for measurement and shortlist of candidate PRO measures will be generated from a systematic search and rapid review of the published literature.[14] The conceptual framework will provide a content map describing the expected relationships between constructs to be measured by the PRO-CAR-T system and the items/domains of a PRO measure.[15] Next, a Delphi review panel involving up to 30 participants will select PRO measures for inclusion in the PRO-CAR-T system from the list of shortlisted measures. The Delphi study will comprise two rounds: 1) an online survey followed by 2) an online workshop to reach consensus on the PRO measures to be included.

For peer review only - http://bmjopen.bmj.com/site/about/guidelines.xhtml

Last, we will hold a series of workshops (or individual interviews depending on participant preference) to: 1) ascertain the essential and desirable components of an ePRO system (co-design) and 2) establish those items from the included PRO measures that, if reported by a CAR-T patient, would be of sufficient clinical concern to require notification to the patient's clinical team. We will explore co-design elements with participants including key stakeholders' requirements for symptom reporting, information, and communication mapped against the patient journey. Findings will ensure the design of the PRO-CAR-T system is grounded in the needs and expectations of users.[16] The co-design approach is based on ISO standards for human-centred design for interactive systems (European Committee for Standardisation 2010), ISPOR guidance on ePRO systems, and the user innovation management method.[16–18] To establish alerting requirements, workshops and interviews will explore stakeholders preferences for the format of alert notifications, establish scoring thresholds and arising actions, and map the end-to-end clinical workflow that will occur within the Atom5™ environment. We will invite up to 30 individuals to take part from the following participant groups: CAR-T patients and their family members, healthcare professionals and academics/researchers.

For each component of intervention development (Work Package 1), we will use descriptive statistics to analyse quantitative survey data (overall and per participant group) using Excel, STATA or SPSS. For qualitative analyses, we will apply thematic analysis to inductively code free text survey responses and workshop transcripts.[19] Two researchers will independently code a subset of the transcripts to cross check the coding strategy and data interpretation and the COREQ checklist will be used as a guideline for reporting qualitative methods and findings.[20] Deductive frameworks will support coding of data to develop the system specification and *a priori* criteria, based on Murphy et al. and Williamson et al. , will be applied to establish consensus on the PRO measures to be included in the PRO-CAR-T digital system.[21,22]

Work Package 2: Usability testing

Usability testing refers to the "formal assessment of the extent to which interaction with a product or system is effective, efficient and perceived as satisfactory by users" (p. 326)[23] and typically involves observation of end users interacting with the digital tool as they complete a series of tasks. CAR T-cell therapy patients and HCPs working in clinical settings offering CAR T-cell therapies will participate in a cognitive interview to evaluate the usability of the PRO-CAR-T system. A sample of 20-30 patients and HCPs will take part in two cycles of testing. The PRO-CAR-T system in Atom5™ will be refined iteratively after each test cycle until no new problems are encountered by participants. The usability test sessions will be conducted remotely using videoconferencing software. Test sessions will be recorded and transcribed verbatim for analysis. During the test session, patient participants will be asked to download Atom5™, access the PRO-CAR-T system, and navigate the system to complete the PRO measures. Whilst navigating the system, patients will be asked to "think aloud" to describe their thought process to the researcher. Patients will be informed that they do not have to answer the

For peer review only - http://bmjopen.bmj.com/site/about/guidelines.xhtml

questions about their symptoms or quality of life truthfully, that the emphasis of testing is to evaluate the PRO-CAR-T system's ease of use from the user perspective, and that any data they enter will not be saved nor used to inform their care. HCPs will login to the PRO-CAR-T clinical dashboard and complete specific tasks (e.g., register a patient, respond to a clinical alert) whilst thinking aloud. In the case of HCPs, fictitious patient data will be used. Upon completing the tasks, participants will be asked to provide feedback relating to their experience using the digital system including ways the user experience could be improved.

A semi-structured topic guide will be used to structure the interview and participants will also complete the System Usability Scale (SUS), a 10-item questionnaire providing an assessment of usability.[24] SUS items are scored on a 5-point rating scale ranging from "strongly disagree" to "strongly agree". Raw scores are converted to 0-100 score with higher scores indicating greater usability. A score greater than 80 is considered evidence of above average user experience.[25] The psychometric properties of the SUS have been evaluated extensively and the scale has been shown to have good internal consistency reliability (Cronbach's alpha = 0.83-0.91) and concurrent validity with other measures of usability.[26] A multi-method approach to usability (i.e., usability metrics, self-completed surveys, and cognitive interviewing techniques) was selected to counter positive recall bias associated with think aloud techniques.[23]

Data analysis will be guided by the Fit Between Individuals, Task and Technology (FITT) framework to describe usability on three dimensions: 1) task-technology fit; 2) individual-technology fit; and 3) individual-task fit.[27] Developed specifically for use in healthcare, the FITT framework enables developers to understand the relationship between users, tasks and technology and the factors affecting optimal device use.[28] Descriptive statistics will be used to describe the demographic characteristics of the study sample, SUS scores, prompt/error rates, and task completion times. Excel and SPSS or STATA statistics software will be used for all statistical analyses.

Cognitive "think aloud" interviews will be qualitatively analysed using thematic analysis and the framework method.[29] A deductive coding framework will be used to code data according to the FITT model and inductive coding will identify additional concepts in the data. NVivo (Version 14) qualitative data analysis software will be used to manage and code the transcribed interview data. Two researchers will independently code a subset of the data to cross check the coding strategy and data interpretation. Problems with the ePRO system identified during usability testing will be logged, discussed with Aparito, and a system change request generated. Refinements to the system will be made before commencing the next test cycle.

Work Package 3: Feasibility study

The PRO-CAR-T feasibility study is a single centre study of the feasibility and acceptability of the new ePRO system when deployed in the UK's National Health Service (NHS). Thirty CAR-T patients will be recruited consecutively to use the PRO-CAR-T system (i.e., in addition to usual care). Patients who consent to participate will be onboarded to the system prior to infusion. During the baseline visit, participants will download the Atom5™ app to their mobile device, give their demographic details

For peer review only - http://bmjopen.bmj.com/site/about/guidelines.xhtml

including age, sex at birth, ethnicity, cancer diagnosis, CAR T-cell therapy, and self-reported experience with technology and complete the PRO measures for the first time. PRO measures will be completed subsequently via the PRO-CAR-T system at pre-specified intervals (measures and frequency of assessments will be established in Work Package 1). We will follow participants for 12-months from date of infusion (Day 0) to understand the feasibility and acceptability of longitudinal collection of PROs for CAR-T patients, their families, and HCPs. The findings will be used to inform a future, definitive randomised controlled trial to assess the effectiveness of remote symptom monitoring using the digital system alongside usual care compared to usual care alone.

Sample size justification

There is no definitive guidance relating to sample sizes in pilot and feasibility studies with a heuristic of 30 (range = 10 to 40) patients generally applied. Therefore, no formal sample size calculation has been performed for the feasibility study. To allow for a 15% attrition rate, a minimum sample of 35 patients will be recruited, allowing recruitment and retention rates to be estimated with 95% confidence interval (CI) maximum widths of 2% and 28%.[30]

Data analysis

Simple descriptive statistics will be reported for each feasibility outcome measure (e.g., number and proportion of eligible participants enrolled). All quantitative analyses will be conducted in SPSS or STATA. Interim analyses will be conducted 6-months after opening the study to recruitment.

Data will be collected from Atom5™ to assess the feasibility and acceptability of the PRO-CAR-T digital platform and *a priori* benchmarks applied:

- *Recruitment*: Number and proportion (%) of eligible patients who consent to take part. The study will be considered feasible if ≥ 50% of eligible patients consent.
- *Retention*: Number and proportion of patients who complete the 12-month PRO assessment. The study will be considered feasible if ≥ 70% of patients complete the final assessment at Month-12.
- *Adherence*: Number and proportion of patients completing the PRO assessments. The study will be considered feasible if ≥ 70% of expected PRO assessments are completed at each timepoint, taking into consideration attrition arising from disease progression, severe toxicity resulting in loss of capacity, or death. We will document reasons for non-completion (e.g., hospital admission). Missed completions will be checked against the patient's medical notes by a member of the clinical team (i.e., research nurse) to identify possible reasons for non-completion (i.e., a record of ICANS or other severe adverse treatment-related events, hospital admission, or death). To be considered adherent, patients will need to have submitted their report via the PRO-CAR-T system within 72 hours of the scheduled timepoint. *Ad hoc* reports will not contribute to the assessment of adherence. Incomplete submissions will be accepted, although missing data is expected to be minimal with the required fields functionality in Atom5™.

For peer review only - http://bmjopen.bmj.com/site/about/guidelines.xhtml

- Number of clinical alerts and number and proportion of patients reporting clinical alerts.
- Number and proportion of patients who withdraw formally from the study and their reasons for withdrawal.
- Actions arising from alerts including number and proportion of patients attending clinic, hospitalisation following clinical alert reporting, estimated time between alert and response by clinical team.
- Number of *ad hoc* PRO assessments completed and the number and proportion of patients submitting *ad hoc* PRO assessments.

At 3-months, 6-months and 12-months post-infusion, acceptability will be assessed using implementation outcome measures: Acceptability of Intervention Measure (AIM), Intervention Appropriateness Measure (IAM), and Feasibility of Intervention Measure (FIM) scales. Psychometric evaluation showed acceptable internal consistency reliability (Cronbach's alpha values ranging from 0.85 – 0.91) and test-retest reliability (Pearson correlation values ranging from 0.73 – 0.88), and a Flesch 5th reading grade level score. Each scale has four items and completion time for all three scales is less than five minutes. Participants will rate their agreement on a Likert scale in response to statements about using the PRO-CAR-T digital system (e.g., "The PRO-CAR-T system is appealing to me.").[31]

Qualitative sub-study

We will conduct a qualitative evaluation of the PRO-CAR-T system with clinicians and a sub-sample of feasibility study participants at approximately 3-months post-infusion. This time interval was selected to ensure patients have sufficient opportunity to use the PRO-CAR-T system. Patients and HCPs who give their consent to participate will take part in an interview lasting up to one hour, with breaks as required. Semi-structured topic guides (tailored to the different participant groups) will be used to ensure key topics are consistently covered. The topic guides will remain flexible and evolve based on findings. We will use reflexive thematic analysis for data analysis in NVivo with two researchers coding a subset of the transcripts to cross check the coding strategy and data interpretation.

PATIENT AND PUBLIC INVOLVEMENT

The Patient and Public Involvement and Engagement (PPIE) plan for the PRO-CAR-T Study sits within the overall BTRU PPIE strategy.[32] The strategy has been developed by patients, carers, members of the public, and researchers who have worked together to agree a PPIE action-plan informed by the UK Standards of Public Involvement in Research.[33] In accordance with principles of co-production and the BTRU PPIE Strategy, researchers will work in partnership with patient and public contributors through a mix of consultation, collaboration and user-led activities.[32] Patient authors have been involved in the co-design of the research protocol, patient and public-facing materials such as lay summaries and patient information leaflets. Future activities will include supporting the interpretation of findings, co-designing engagement strategies and materials to share findings with the public (e.g., lay summaries, press releases, creative and social media communications), co-authoring

For peer review only - http://bmjopen.bmj.com/site/about/guidelines.xhtml

academic/clinical outputs, and supporting Research Governance activities (e.g., patients and public contributors are included in the BTRU Project Management Group).

ETHICS AND DISSEMINATION

Ethical approval for the study was provided by the UK's Health Research Authority (HRA) Social Care Research Ethics Committee B Proportionate Review Subcommittee (HSC REC B) (Ref: 23/NI/0104). The results of the study will be published in a peer-reviewed journal, presented at conferences and symposia and shared with patients and members of the public through lay summaries via multiple media.

ACKNOWLEDGMENTS
We thank the Blood and Transplant Research Unit in Precision Cellular Therapeutics Patient and Public Involvement (PPIE) Group for their help and support in developing the protocol for the PRO-CAR-T Study.

Data Sharing: No additional data available.

Contributors: MC, OLA, SEH, DB, CC, FrK, AD, EHD designed the study; SEH developed the first draft of the protocol. DB, PF, FrK, AD, CC provided clinical expertise; MC, SEH, CM, OLA, KS, FK, EDH provided methodological expertise; LP, JA, EC, JRA and the BTRU PPIE group provided patient input. All investigators provided advice and critical input to the study design. All authors provided critical review of the manuscript and approved the final version.

Funding: This study is sponsored by the University of Birmingham and funded by the National Institute for Health and Care Research (NIHR) Blood and Transplant Research Unit (BTRU) in Precision Cellular Therapeutics (Reference: NIHR 203339). The views expressed are those of the author(s) and not necessarily those of the NIHR or the Department of Health and Social Care.

Competing interests: MC is Director of the Birmingham Health Partners Centre for Regulatory Science and Innovation, Director of the Centre for the Centre for Patient Reported Outcomes Research and is a National Institute for Health Research (NIHR) Senior Investigator. MC receives funding from the National Institute for Health Research (NIHR), UK Research and Innovation (UKRI), NIHR Birmingham Biomedical Research Centre, the NIHR Surgical Reconstruction and Microbiology Research Centre, NIHR ARC West Midlands, UK SPINE, European Regional Development Fund – Demand Hub and Health Data Research UK at the University of Birmingham and University Hospitals Birmingham NHS Foundation Trust, Innovate UK (part of UKRI), Macmillan Cancer Support, UCB Pharma, Anthony Nolan, GSK and Gilead. MJC has received personal fees from Astellas, Aparito Ltd, CIS Oncology, Takeda, Merck, Daiichi Sankyo, Glaukos, GSK and the Patient-Centered Outcomes Research Institute (PCORI) outside the submitted work. In addition, a family member owns shares in GSK. **OLA** receives funding from the NIHR Birmingham BRC, West Midlands, Birmingham, NIHR ARC West Midlands, NIHR BTRU, UKRI, Health Foundation, Anthony Nolan, Gilead Sciences Ltd, Sarcoma UK, and GSK. He declares personal fees from Gilead Sciences Ltd, Merck and GSK outside the submitted work. **SEH** receives funding from the NIHR, UKRI, Rinri Therapeutics, Anthony Nolan, and declares personal fees from Cochlear Ltd, Astra Zeneca, Pfizer, CIS Oncology, and Aparito Ltd outside the submitted work. **CM** receives funding from NIHR Surgical Reconstruction and Microbiology Research Centre (SRMRC), UKRI, Anthony Nolan, and declares personal fees from Aparito Ltd outside the submitted work. All other authors declare no competing interests.

Patient consent for publication: Not required.

Provenance and peer review: Not commissioned; peer reviewed for ethical and funding approval prior to submission.

For peer review only - http://bmjopen.bmj.com/site/about/guidelines.xhtml

Open access: This is an open access article distributed in accordance with the Creative Commons Attribution 4.0 International (CC BY 4.0) licence, which permits others to copy, redistribute, remix, transform and build upon this work for any purpose, provided the original work is properly cited, a link to the licence is given, and indication of whether changes were made. See: https://creativecommons.org/ licenses/by/4.0/.

ORCiDs

Sarah E Hughes: 0000-0001-5656-1198

Olalekan L Aiyegbusi: 0000-0001-9122-8251

Christel McMullan: 0000-0002-0878-1513

Karen Shaw: 0000-0001-7120-8595

Alastair Denniston: 0000-0001-7849-0087

Francesca Kinsella: 0000-0002-6022-2567

Foram Khasturia: 0009-0004-3076-0593

Elin Haf Davies: 0000-0002-4987-0088

Charles Craddock: 0000-0001-5041-6678

Melanie Calvert 0000-0002-1856-837X

REFERENCES

1 Grigor EJM, Fergusson D, Kekre N, *et al.* Risks and Benefits of Chimeric Antigen Receptor T-Cell (CAR-T) Therapy in Cancer: A Systematic Review and Meta-Analysis. *Transfus Med Rev*. 2019;33:98–110.

2 Neelapu SS, Tummala S, Kebriaei P, *et al.* Toxicity management after chimeric antigen receptor T cell therapy: one size does not fit "ALL." *Nat Rev Clin Oncol*. 2018;15:218.

3 Santomasso B, Bachier C, Westin J, *et al.* The Other Side of CAR T-Cell Therapy: Cytokine Release Syndrome, Neurologic Toxicity, and Financial Burden. *American Society of Clinical Oncology Educational Book*. 2019;433–44.

4 Whisenant MS, Srour SA, Williams LA, *et al.* The Unique Symptom Burden of Patients Receiving CAR T-Cell Therapy. *Semin Oncol Nurs*. 2021;37:151216.

5 Brudno JN, Kochenderfer JN. Toxicities of chimeric antigen receptor T cells: recognition and management. *Blood*. 2016;127:3321–30.

6 Oswald LB, Li X, Carvajal R, *et al.* Longitudinal Collection of Patient-Reported Outcomes and Activity Data during CAR-T Therapy: Feasibility, Acceptability, and Data Visualization. *Cancers*. 2022;14:2742. doi: 10.3390/cancers14112742

7 US Food and Drug Administration. Principles for Selecting, Developing, Modifying, and Adapting Patient-Reported Outcome Instruments for Use in Medical Device Evaluation: Draft Guidance for Industry and Food and Drug Administration Staff, And Other Stakeholders. 2020.

8 Basch E, Deal AM, Kris MG, *et al.* Symptom monitoring with patient-reported outcomes during routine cancer treatment: A randomized controlled trial. *Journal of Clinical Oncology*. 2016;34:557–65.

For peer review only - http://bmjopen.bmj.com/site/about/guidelines.xhtml

9 Atkinson TM, Ryan SJ, Bennett AV, *et al.* The Association between Clinician-Based Common Terminology Criteria for Adverse Events (CTCAE) and Patient-Reported Outcomes (PRO): A Systematic Review. *Support Care Cancer*. 2016;24:3669–76.

10 Calvert M, Kyte D, Price G, *et al.* Maximising the impact of patient reported outcome assessment for patients and society. *BMJ*. 2019;364. doi: 10.1136/bmj.k5267

11 Greenhalgh J, Gooding K, Gibbons E, *et al.* How do patient reported outcome measures (PROMs) support clinician-patient communication and patient care? A realist synthesis. *Journal of Patient-Reported Outcomes*. 2018;2. doi: 10.1186/s41687-018-0061-6

12 Efficace F, Cannella L, Sparano F, *et al.* Chimeric Antigen Receptor T-cell Therapy in Hematologic Malignancies and Patient-reported Outcomes: A Scoping Review. *HemaSphere*. 2022;6:e802.

13 UK Stem Cell Strategic Forum. A ten year vision for stem cell transplantation and cellular therapies. 2022.

14 Khatsuria F, Calvert M, McMullan C, *et al.* Protocol for a rapid review to inform concept elicitation and measure selection for an electronic patient-reported outcome (ePRO) system for patients with haematological malignancies receiving CAR-T cellular therapies. Published Online First: 12 May 2023. doi: 10.17605/OSF.IO/A6QBX

15 Donatti C, Wild D, Hareendran A. The use of conceptual models, conceptual frameworks, and endpoint models to support label claims of treatment benefit using patient reported outcomes. *ISPOR Connections*. ;9–12.

16 Nielsen AC, Rotger-Griful S, Kanstrup AM, *et al.* User-Innovated eHealth Solutions for Service Delivery to Older Persons With Hearing Impairment. *Am J Audiol*. 2018;27:403–16.

17 Zbrozek A, Hebert J, Gogates G, *et al.* Validation of Electronic Systems to Collect Patient-Reported Outcome (PRO) Data—Recommendations for Clinical Trial Teams: Report of the ISPOR ePRO Systems Validation Good Research Practices Task Force. *Value in Health*. 2013;16:480–9.

18 Kanstrup AM, Bertelsen PS. User Innovation Management: A Handbook. 2011.

19 Braun V, Clarke V. *Thematic analysis: a practical guide*. London: SAGE Publications 2022.

20 Tong A, Sainsbury P, Craig J. Consolidated criteria for reporting qualitative research (COREQ): a 32-item checklist for interviews and focus groups. *International Journal for Quality in Health Care*. 2007;19:349–57.

21 Murphy M, Hollinghurst S, Salisbury C. Agreeing the content of a patient-reported outcome measure for primary care: a Delphi consensus study. *Health Expectations*. 2017;20:335–48.

22 Williamson PR, Altman DG, Bagley H, *et al.* The COMET Handbook: version 1.0. *Trials*. 2017;18:280.

23 Aiyegbusi OL. Key methodological considerations for usability testing of electronic patient-reported outcome (ePRO) systems. *Qual Life Res*. 2020;29:325–33.

24 Brooke J. SUS: A quick and dirty usability scale. *Usability Eval Ind*. 1995;189.

25 Lewis JR, Sauro J. Item benchmarks for the system usability scale. *Journal of Usability Studies*. 2018;13.

26 Lewis JR. The System Usability Scale: Past, Present, and Future. *International Journal of Human–Computer Interaction*. 2018;34:577–90.

27 Ammenwerth E, Iller C, Mahler C. IT-adoption and the interaction of task, technology and individuals: a fit framework and a case study. *BMC Medical Informatics and Decision Making*. 2006;6:3.

For peer review only

For peer review only - http://bmjopen.bmj.com/site/about/guidelines.xhtml

28 Prgomet M, Georgiou A, Callen J, *et al.* Fit Between Individuals, Tasks, Technology, and Environment (FITTE) Framework: A Proposed Extension of FITT to Evaluate and Optimise Health Information Technology Use. *MEDINFO 2019: Health and Wellbeing e-Networks for All*. IOS Press 2019:744–8. https://doi.org/10.3233/SHTI190322

29 Gale NK, Heath G, Cameron E, *et al.* Using the framework method for the analysis of qualitative data in multi-disciplinary health research. *BMC Med Res Methodol*. 2013;13:117.

30 Herzog M. Considerations in Determining Sample Size for Pilot Studies. *Research in Nursing & Health*. 2008;31:180–91.

31 Weiner BJ, Lewis CC, Stanick C, *et al.* Psychometric assessment of three newly developed implementation outcome measures. *Implementation Science*. 2017;12:108.

32 NIHR BTRU in Precision Cellular Therapeutics. NIHR BTRU in Precision Cellular Therapeutics. Patient and Public Involvement and Engagement Strategy, 2022-2027. 2022.

33 UK Standards for Public Involvement. Better public involvement for better health and social care research. 2019. https://sites.google.com/nihr.ac.uk/pi-standards/home

For peer review only

For peer review only - http://bmjopen.bmj.com/site/about/guidelines.xhtml

For peer review only

For peer review only - http://bmjopen.bmj.com/site/about/guidelines.xhtml

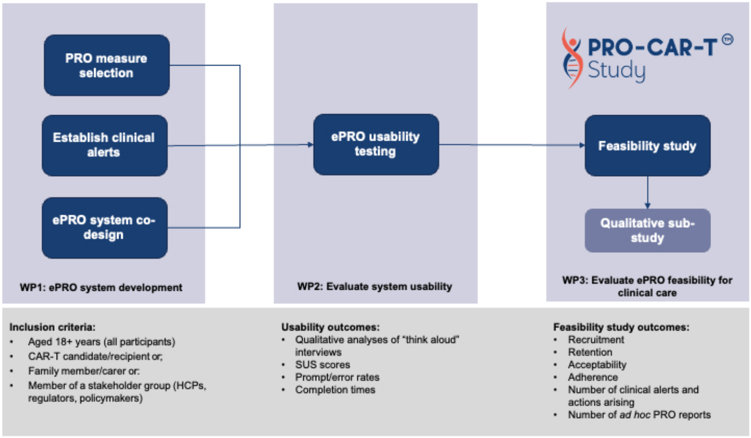

Figure 1. Study flow diagram for the PRO-CAR-T Study. SUS, System Usability Scale; HCP, Healthcare Professional; CAR-T, Chimeric Antigen Receptor T-cell; ePRO, electronic patient-reported outcome; PRO, patient-reported outcome.

338x190mm (72 x 72 DPI)
