# Supplementary material for: Protocol for a mixed-methods study to develop and feasibility test a digital system for the capture of patient-reported outcomes (PROs) in patients receiving chimeric antigen receptor T-cell (CAR-T) therapies (the PRO-CAR-T study)
Source: BMJ Open. 2024 Mar 29;14(3):e085392. doi: 10.1136/bmjopen-2024-085392 (PMC10982800; doi:10.1136/bmjopen-2024-085392)
Supplement: Reviewer comments [file bmjopen-2024-085392.reviewer_comments.pdf]

PEER REVIEW HISTORY

BMJ Open publishes all reviews undertaken for accepted manuscripts. Reviewers are asked to complete a checklist review form (**http://bmjopen.bmj.com/site/about/resources/checklist.pdf**) and are provided with free text boxes to elaborate on their assessment. These free text comments are reproduced below.

ARTICLE DETAILS

**TITLE (PROVISIONAL)**	Protocol for a mixed-methods study to develop and feasibility test a digital system for the capture of Patient-Reported Outcomes (PROs) in patients receiving Chimeric Antigen Receptor T-cell (CAR-T) therapies (The PRO-CAR-T Study)
**AUTHORS**	Hughes, Sarah; McMullan, Christel; Aiyegbusi, Olalekan Lee; Shaw, Karen; Kinsella, Francesca; Ferguson, Paul; Khatsuria, Foram; Burns, David; Pyatt, Lester; Ansell, John; Chakera, Evelyn; Richardson-Abraham, Julie; Denniston, Alastair; Davies, Elin Haf; Craddock, Charles; Calvert, Melanie

This article was not externally reviewed at BMJ Open. Protocols that have been independently assessed prior to submission to BMJ Open are usually fast-tracked to publication on the grounds that further substantial changes will not be possible. This independent assessment will usually be external, independent review for both a substantial grant award from a non-commercial or government funder and ethics approval.
